# Early poststroke clinically significant fatigue predicts functional independence: a prospective longitudinal study

**DOI:** 10.3389/fneur.2024.1364446

**Published:** 2024-06-11

**Authors:** Alan Juárez-Belaúnde, Vanesa Soto-León, Michele Dileone, Elena Orcajo, Natacha León-Álvarez, Alberto Muñoz, Jesus Tornero, Antonio Oliviero

**Affiliations:** ^1^Advanced Neurorehabilitation Unit, Hospital Los Madroños, Madrid, Spain; ^2^FENNSI Group, Hospital Nacional de Parapléjicos, SESCAM, Toledo, Spain; ^3^Neurology Department, Hospital Nuestra Señora del Prado, SESCAM, Talavera de la Reina, Spain; ^4^Department of Radiology, Faculty of Health Sciences, UCLM, Talavera de la Reina, Spain; ^5^School of Medicine, Universidad Complutense de Madrid, Madrid., Spain

**Keywords:** ischemic stroke, hemorrhagic stroke, fatigue, FSS, NIHSS, outcome

## Abstract

**Background:**

Poststroke fatigue is a prevalent issue among stroke survivors, significantly impeding functional recovery and diminishing their quality of life.

**Aim:**

This prospective cohort study aims to investigate the association between poststroke fatigue and the extent of functional recovery in survivors of ischemic and hemorrhagic strokes. Additionally, it seeks to delineate the temporal progression of poststroke fatigue in these two stroke subtypes.

**Methods:**

We assessed a cohort of 79 patients recovering from acute ischemic or hemorrhagic strokes. Poststroke fatigue was quantified using the Fatigue Severity Scale (FSS) and the Numeric Rating Scale (NRS_fatigue_). Patients’ condition was evaluated using the National Institute of Health Stroke Scale (NIHSS), and functional independence levels were determined using the Barthel Index for Activities of Daily Living (BIADL) and the Modified Rankin Scale (MRS). Depressive mood and pain were measured using the Beck Depression Inventory (BDI) and the Numeric Rating Scale for pain (NRSpain), respectively.

**Results:**

Our primary findings indicate that the early manifestation of clinically significant fatigue (CSF) is predictive of a poorer trajectory in functional independence levels during recovery. Furthermore, we observed differing patterns of fatigue progression between ischemic and hemorrhagic strokes. Fatigue tends to ameliorate over time in hemorrhagic stroke cases, paralleling functional recovery, while it remains stable over time in ischemic stroke cases.

**Conclusion:**

Our results underscore the detrimental impact of early poststroke fatigue on long-term outcomes. Furthermore, they highlight the imperative of managing poststroke fatigue, particularly during the subacute phase of stroke recovery.

## 1 Introduction

Stroke poses a significant public health concern, with approximately 60–70% of individuals unable to resume their previous lifestyle after stroke (1). Poststroke fatigue (PSF) emerges as a common symptom following stroke, with reported prevalence ranging between 23 and 85% in the literature (2, 3). Described as early exhaustion accompanied by weariness, lack of energy, and reluctance to exertion, poststroke fatigue persists during physical or mental activity and often remains unaffected by rest (2–5). This fatigue detrimentally impacts quality of life and workability and is even linked to a heightened mortality risk (6–8). Despite its profound implications, poststroke fatigue receives relatively little attention, with no specific treatments currently available (9).

When a stroke occurs, it can induce profound shifts in the brain’s function and structure. It disrupts the delicate balance of neural networks. Moreover, the immune system’s response to a stroke can also exacerbate fatigue. As part of the body’s natural response to injury, an inflammatory process is activated, potentially flooding the system with inflammatory cytokines, fostering a protective response but also contributing to a pervasive sense of exhaustion (2, 10, 11). Emotional well-being is compromised following a stroke, with depressive mood, anxiety, and stress frequently taking hold. These psychological states are closely entwined with fatigue. Moreover, cognitive efforts become disproportionately taxing after a stroke and the strenuous journey of adjusting to new physical and cognitive limitations demands substantial mental exertion, which can compound feelings of fatigue (12, 13). Disorders such as sleep apnoea and insomnia, which are prevalent in stroke survivors, together with the sleep changes due to hospitalization can impair the restorative quality of sleep, leaving individuals grappling with fatigue during the daylight hours. Furthermore, a reduction in physical activity after a stroke is another contributing factor. It can result in a decline in muscle strength, intensifying the sensation of fatigue. Hitherto, the body’s metabolic and hormonal milieu may be altered by a stroke, leading to shifts in energy levels and influencing how fatigue is perceived (12, 13).

The complexity of PSF is further amplified by changes in neuroplasticity after a stroke, which can affect the excitability of cortical neurons. An imbalance may arise between excitatory and inhibitory signals in the brain, either ramping up neural activity or causing a lull, each contributing to fatigue in its own way (2, 11).

The connectivity between different brain regions may be reshaped, potentially altering the efficiency with which these areas communicate. Such changes are thought to impact the personal experience of fatigue (2, 11).

It is important to note that these mechanisms are not mutually exclusive and can interact in complex ways to contribute to the experience of poststroke fatigue. Furthermore, individual differences in genetics, pre-stroke health status, and the location and severity of the stroke can influence the development and severity of PSF. Ongoing research is aimed at better understanding these mechanisms to improve the management and treatment of poststroke fatigue. After a stroke, the brain undergoes a range of neuroplastic changes as it attempts to recover and reorganize its functions. These changes can affect the excitability of cortical neurons, potentially leading to alterations in how signals are transmitted across neural networks. It is thought that in some individuals, these alterations might contribute to the onset or exacerbation of fatigue symptoms.

PSF can significantly interfere with the rehabilitation process for stroke survivors. Its impact may affect not only the physical aspect of recovery but also cognitive and emotional well-being. Fatigue can decrease a patient’s ability to actively participate in rehabilitation sessions. Patients may find it difficult to complete exercises or engage in activities for the duration required to achieve optimal outcomes. This reduced participation can slow progress and decrease the overall effectiveness of the rehabilitation program (4, 14). PSF is not just physical but also cognitive. Patients experiencing cognitive fatigue may have difficulty focusing, remembering instructions, and performing tasks that require cognitive effort. This can interfere with cognitive rehabilitation efforts and impede recovery in cognitive functions such as memory, attention, and executive functions. Fatigue can exacerbate psychological conditions such as depressive mood and anxiety, which are common after a stroke (4, 13, 14). These conditions can further reduce motivation and engagement in rehabilitation. Moreover, the frustration of dealing with persistent fatigue can lead to decreased morale and motivation, critical components of successful rehabilitation treatments.

The recent consensus among experts underscores the imperative of early recognition of poststroke fatigue in stroke patients, advocating for intervention targeting modifiable factors. Key priorities include employing appropriate tools for clinical identification and examination, elucidating its pathophysiology, and exploring therapeutic avenues such as neuromodulation, therapeutic exercise, psychoeducation, and pharmacology (15).

To the best of our knowledge, the association between poststroke fatigue in the early stages poststroke and functional outcomes remains inadequately explored (5). Given its potential to impede engagement in physical and social activities and rehabilitation, this aspect assumes paramount importance (16–18). A recent retrospective study highlights the correlation between poststroke fatigue at admission and functional outcomes (19).

In this prospective study, we investigate the impact of initial poststroke fatigue on the functional recovery of stroke survivors enrolled in a neurorehabilitation program. Specifically, we assess the trajectory of fatigue over the first 6 months and examine the influence of stroke subtype (ischemic or hemorrhagic).

## 2 Methods

### 2.1 Study design

We designed a prospective cohort study: we recruited first-ever stroke patients admitted to the Advanced Neurorehabilitation Unit of the “Los Madroños” Hospital. We evaluated patients using clinical scales, fatigue scales, and functional and quality of life scales at admission and at 3 and 6 months since admission. During this time, patients underwent a neurorehabilitation process that included physiotherapy, occupational therapy, speech therapy, and neuropsychology, according to the individual neurological condition of each patient. Cognitive functions were screened using the Mini-Mental State Evaluation (MMSE). The study was approved by the Ethics Committee (Hospital Universitario Severo Ochoa, Madrid, ref.: A1434). Participants provided informed written consent complying with the norms of the Declaration of Helsinki.

### 2.2 Participants

The inclusion criteria were as follows: (1) age ≥ 18 and ≤ 85; (2) definite clinical diagnosis of first-ever stroke with neuroradiological confirmation; (3) ability to participate in the rehabilitation program as evaluated by an expert physician; (4) possibility of obtaining informed consent. The exclusion criteria were as follows: (1) neurological disorders other than first-ever stroke (including spinal cord injury of vascular origin and previous stroke); (2) severe psychiatric disorder; (3) MMSE <23; (4) Severe communication difficulties such as aphasia; (5) Medical disorders that may account for severe fatigue (e.g., severe liver and kidney disease, severe cardiopulmonary disorders, etc.).

Ninety-five patients were recruited and screened, and sixteen were excluded due to various reasons, including loss of follow-up due to death, new stroke occurrence during the study, severe comorbidity from COVID-19 infection, withdrawal of consent, or being lost to follow-up and 79 patients entered the final analysis.

### 2.3 Outcome measures

Demographic and clinical data were collected during inpatient stay at baseline (T0) and at 3 (T1) and 6 (T2) months after admission, with patients continuing neurorehabilitation in a hospital setting. Demographic and clinical variables collected included age, sex, number of days from stroke onset to admission to the neurorehabilitation program, and type of stroke (ischemic or hemorrhagic). Fatigue was measured using the Fatigue Severity Scale (FSS) and the Numeric Rating Scale (NRS_fatigue_, 0–10 scale, where 0 is the absence of fatigue and 10 is extreme fatigue). We defined clinically significant fatigue (CSF) in individuals with FSS ≥4. In three patients, fatigue evaluation cannot be obtained at baseline, and for this reason, we used the first fatigue evaluation available. Stroke severity and functional independence levels were rated using the National Institute of Health Stroke Scale (NIHSS), Barthel Index for Activities of Daily Living (BIADL), and Modified Rankin Scale (MRS). Depressive mood and pain were evaluated using the Beck Depression Inventory (BDI) and the Numeric Rating Scale (NRS_pain_) respectively.

### 2.4 Data analysis

#### 2.4.1 Baseline characteristics

Continuous variables are represented as mean and standard deviation (SD), and non-parametrical data are represented by median and range.

At baseline (T0), we used an unpaired *t*-test for parametric variables and the Mann–Whitney U test for non-parametric variables to compare the ischemic and hemorrhagic stroke groups. Since this comparison showed some difference (see results section) at baseline, the time course of the main variables was separately analyzed for ischemic and hemorrhagic stroke groups.

### 2.5 Correlation analysis for data reduction

#### 2.5.1 FSS and NRS_fatigue_

To test the association of fatigue measured with the FSS and the NRS_fatigue_, we performed the Spearman test between FSS and NRS_fatigue_. As these variables were strongly correlated (see Results section), we considered them to provide the same information, and therefore, we included only the FSS in further analysis. This decision follows recommendations for using the FSS due to its high internal consistency, stability over time, sensitivity to clinical changes, and correlation with other fatigue scales (10).

#### 2.5.2 FSS, BDI, and NRS_pain_

To test the association of fatigue with depressive mood and pain, we performed the Spearman test between the FSS at baseline and BDI and NRS_pain_. As these variables were strongly correlated (see Results section), we considered them a symptom cluster and did not analyze them separately (see Discussion section) (19, 20).

#### 2.5.3 Time-course of the NIHSS, BIADL, MRS, and FSS

The data were normalized by dividing each individual value by the baseline mean of its respective group. This normalization was performed separately for the ischemic and hemorrhagic groups. This method ensured that the baseline (T0) was normalized to 1 while still allowing for estimation of the variance. Moreover, T1 and T2 can be easily represented and analyzed as change from the baseline (T0). As normal distribution was not achieved, all data were analyzed as non-parametric. The non-parametric Friedman test for repeated measures was used to evaluate the changes between the three-time points studied (each variable was independently evaluated). When significant, paired *post-hoc* comparisons were made using the conover test (bonferroni corrected, planned comparisons *n* = 3; T0vsT1, T0vsT2, and T1vsT2).

#### 2.5.4 Confirmatory analysis

Regarding the FSS, which showed a different time course between the ischemic and hemorrhagic stroke groups (as detailed in the Results section), the normalized data of these groups were compared using both frequentist and Bayesian approaches. For the frequentist approach, we used the Mann–Whitney U test with Bonferroni correction, conducting planned comparisons (*n* = 2; T1_ischemic_ vs. T1_hemorrhagic_, T2_ischemic_ vs. T2_hemorrhagic_). For the Bayesian approach, we used the Bayesian Mann–Whitney U test, which was based on 5 Markov chains with 1.000 iterations each.

#### 2.5.5 Predictive value of CSF on functional recovery and outcome: binary logistic regression analysis

To test the effects of poststroke fatigue on functional recovery and outcome 6 months after stroke, we performed a binary logistic regression analysis including both ischemic and hemorrhagic stroke patients. The binary dependent variable was the presence or not of clinically significant fatigue (CSF, e.g., FSS ≥4) at baseline. The covariate variables were the ratio of NIHSS, BIADL, and MRS at T2 (individual data at T2/baseline mean; the ischemic and hemorrhagic groups were normalized to their respective means) that were independently analyzed. All these analyses incorporated the normalized NIHSS at baseline to correct for initial severity. The group was included in the analysis as an independent variable. All the statistically significant models (*p* < 0.05) were used for further analyses.

All the statistical tests were performed using JASP (version 0.16.3), and all *p*-values <0.05 were considered significant.

## 3 Results

### 3.1 Baseline characteristics

The demographic and clinical data and statistics are reported in Table 1. To summarize, the hemorrhagic group had a more severe neurological deficit than the ischemic group. The two groups were similar in the other variables with a tendency for the hemorrhagic group to have higher levels of fatigue. The neuroimaging data are also reported in Table 1, using the “Oxfordshire Community Stroke Project classification.” Additionally, we provided the number of stroke patients with mainly hemispheric or non-hemispheric strokes.

**Table 1:** Stratification using the Oxfordshire Community Stroke Project classification at baseline.

Variable	All	Ischemic	Hemorrhagic	*p*-value
*N*	79	48	31	-
**Demographic**				
AGE (years, mean (SD))	67.4 (9.9)	67.7 (9.5)	66.8 (10.6)	0.682*
SEX (Male/Female)	52/27	32/16	20/11	0.844**
**Clinical characteristics**				
FSS (median (range))	3.2 (6.0)	2.9 (5.2)	4.2 (6.0)	0.117***
NRS_fatigue_ (median (range))	4.0 (10.0)	3.5 (10.0)	5.0 (10.0)	0.192***
CSF	33 (42%)	16 (33%)	17 (55%)	0.058**
NIHSS (median (range))	10.0 (25.0)	7.5 (23.0)	15.0 (25.0)	0.002***
BIADL (median (range))	25.0 (100.0)	30.0 (100.0)	15.0 (100.0)	0.002***
MRS (median (range))	5.0 (4.0)	4.0 (4.0)	5.0 (4.0)	0.007***
BDI (median (range))	11.0 (49.0)	11.5 (48.0)	11.0 (49.0)	0.948***
NRS_pain_ (median (range))	0.0 (10.0)	0.0 (10.0)	1.0 (10.0)	0.219***
Onset to admission (days, mean (SD))	26.6 (28.6)	26.2 (29.3)	27.2 (28.0)	0.884*
**Neuroimaging characteristics**				
Hemispheric	*N* = 65 (82%)	*N* = 35 (73%)	*N* = 30 (97%)	
Non-hemispheric	*N* = 14 (18%)	*N* = 13 (27%)	*N* = 1 (3%)	
TACI		*N* = 3 (6,3%)		
PACI		*N* = 16 (33,3%)		
POCI		*N* = 13 (27,1%)		
LACI		*N* = 16 (33,3%)		

*N*: number of participants; SD: Standard Deviation; FSS: Fatigue Severity Scale; NRS: Numeric Rating Scale; CFS: Clinically Significant Fatigue (FSS > 4); NIHSS: National Institute of Health Stroke Scale; BIADL: Barthel index for Activities of Daily Living; MRS: Modified Rankin Scale; BDI: Beck Depression Inventory; MAS: Modified Ashworth Scale; TACI: total anterior circulation infarct; PACI: partial anterior circulation infarct; POCI: posterior circulation infarct; LACI: lacunar infarct. * = *t* test; ** = chi-square test; *** = Mann–Whitney U test. Bold indicates significant *p* values.

### 3.2 Correlation analysis

#### 3.2.1 FSS and NRS_fatigue_

A strong association between the FSS and the NRS_fatigue_ was observed using the Spearman test (rho = 0.620, *p* < 0.001). As these variables strongly correlated, we considered them to provide the same information and included only the FSS in further analysis.

#### 3.2.2 FSS, BDI, and NRS_pain_

A strong association between the FSS and depressive mood (BDI) and NRS_pain_ was observed using the Spearman test (FFS and BDI, rho = 0.434, *p* < 0.001; FSS and NRS_pain_, rho = 0.318, *p* = 0.004; BDI and NRS_pain_, rho = 0.345, *p* = 0.002). Since these variables exhibited strong correlation, we considered them as a symptom cluster and did not analyze them separately (see discussion section). We included only the FSS in the further analysis (20).

#### 3.2.3 Time-courses of the NIHSS, BIADL, MRS and FSS

**Figure 1:**
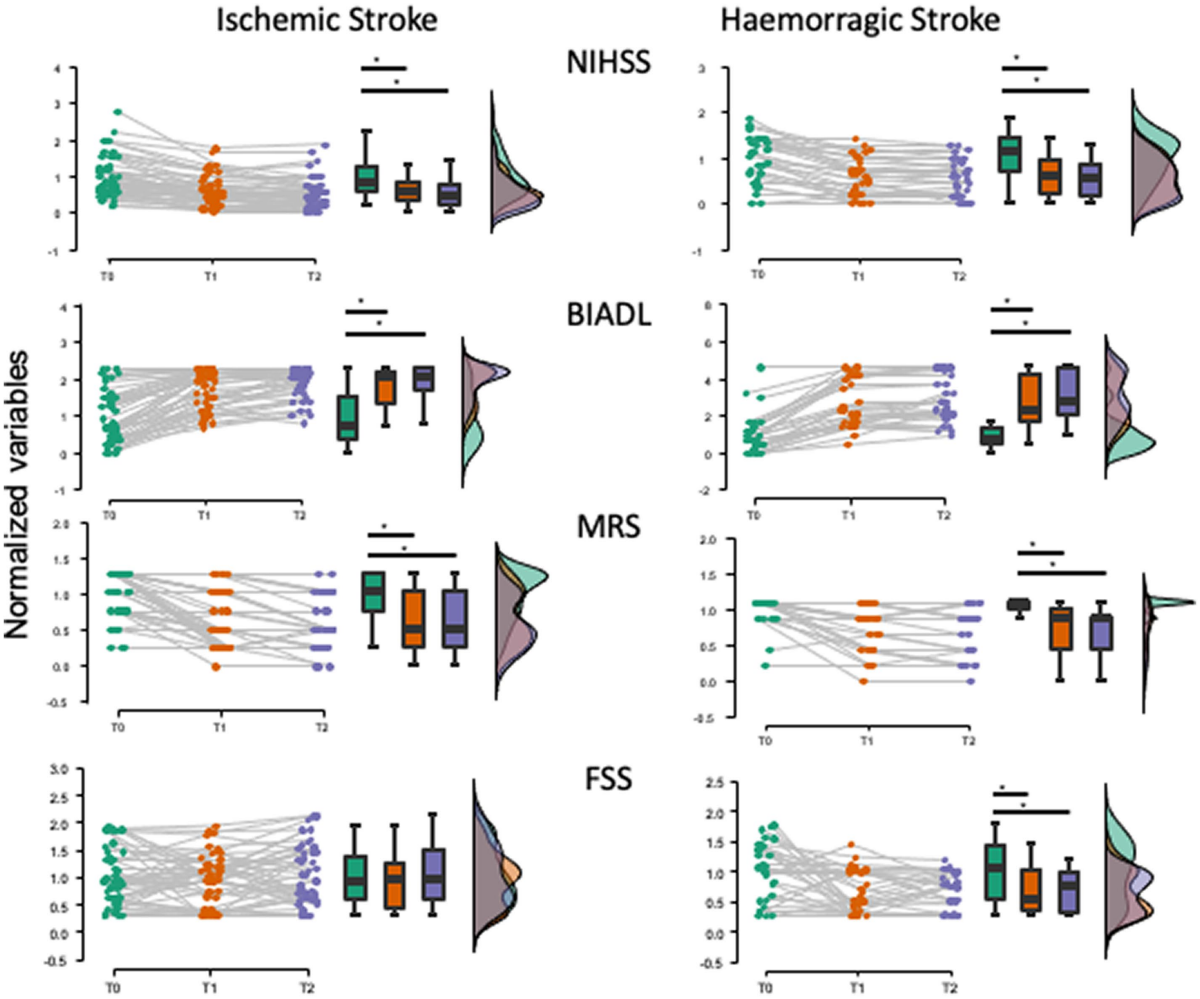
Time courses of NIHSS, BIADL, MRS, and FSS in the ischemic and hemorrhagic stroke groups. Raincloud plots show the data normalized to the baseline mean for each group. The time course of FSS in hemorrhagic stroke is different from that in ischemic stroke, with the FSS values decreasing significantly over time in the hemorrhagic group. Variables measured at baseline (T0) and at 6 (T1) and 12 (T2) months after stroke. * *p* < 0.05.

The course of the NIHSS, BIADL, MRS, and FSS are displayed in Figure 1.

#### 3.2.4 Ischemic stroke

The Friedman test showed a significant effect of TIME on the NIHSS (χ^2^_(2)_ = 59.482, *p* < 0.001), BIADL (χ^2^_(2)_ = 6.176, *p* < 0.001), and MRS (χ^2^_(2)_ = 56.876, *p* < 0.001). *Post-hoc* analyses showed that these variables significantly improved when compared to baseline both at 3 (T1) and at 6 (T2) months after stroke (conover, all p_bonf_ < 0.001). The FSS was unchanged over time (χ^2^_(2)_ = 1.266, *p* = 0.531).

According to the FSS score, poststroke CSF was present in 16 out of 48 patients (33%) at baseline (T0), in 12 out of 48 patients (25%) at 3 months (T1), and in 19 out of 48 patients (39%) at 6 months (T2) after stroke.

#### 3.2.5 Hemorrhagic stroke

The Friedman test showed a significant effect of TIME on the NIHSS (χ2 (2) = 35.154, *p* < 0,001), BIADL (χ2 (2) = 49.929, *p* < 0,001), and MRS (χ2 (2) = 27.835, *p* < 0,001) *Post-hoc* analyses showed that these variables significantly improved when compared to baseline both at 3 (T1) and at 6 (T2) months after stroke (Conover, all p_bonf_ < 0.001). In this group, the FSS also improved over time (χ2 (2) = 9.768, *p* = 0.008). The *post-hoc* analyses showed that these variables significantly improved when compared to baseline both at 3 (T1) and at 6 (T2) months after stroke (Conover, p_bonf_ < 0.0024). According to the FSS score, poststroke CSF was present in 17 out of 31 patients (55%) at baseline (T0), in 9 out of 31 patients (29%) at 3 months (T1), and in 5 out of 31 patients (16%) at 6 months (T2) after stroke.

#### 3.2.6 Confirmatory analysis

**Figure 2:**
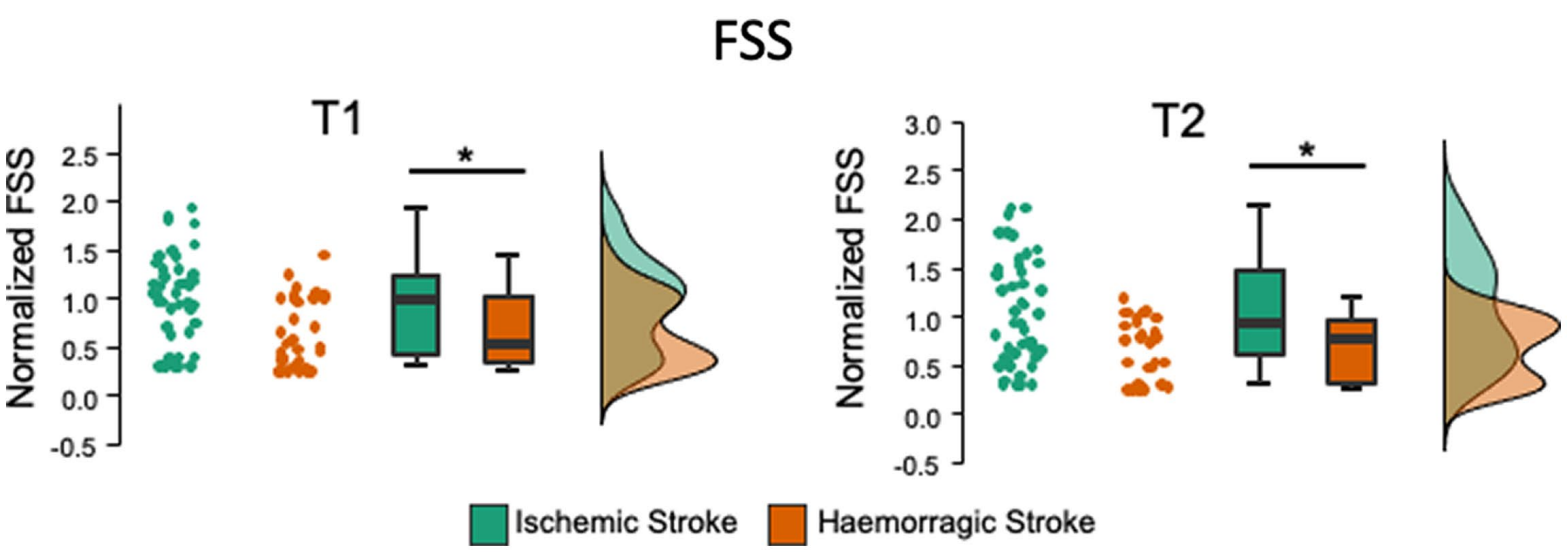
Comparison of the FFS between the ischemic and hemorrhagic stroke groups at 6 (T1) and 12 (T2) months after stroke. The FSS in hemorrhagic stroke is significantly lower at T1 (with moderate evidence) and T2 (strong evidence). Raincloud plots show the data normalized to the baseline mean for each group. * *p* < 0.05; 3 < BF moderate evidence; 10 < BF strong evidence; BF Bayes Factor.

The frequentist analysis using the Mann–Whitney U test showed a significant reduction in the FSS at both T1 (*p* < 0.005) and T2 (*p* < 0.003) in the hemorrhagic group compared to the ischemic group (Figure 2). These comparisons were also confirmed using the Bayesian approach. The Bayesian analysis confirmed that the data provide moderate evidence for the reduction of the FSS at T1(BF_10_ = 9.7) and strong evidence at T2 (BF_10_ = 17.5) in the hemorrhagic group compared to the ischemic group (Figure 2).

#### 3.2.7 Predictive value of CSF on functional recovery and outcome

**Figure 3:**
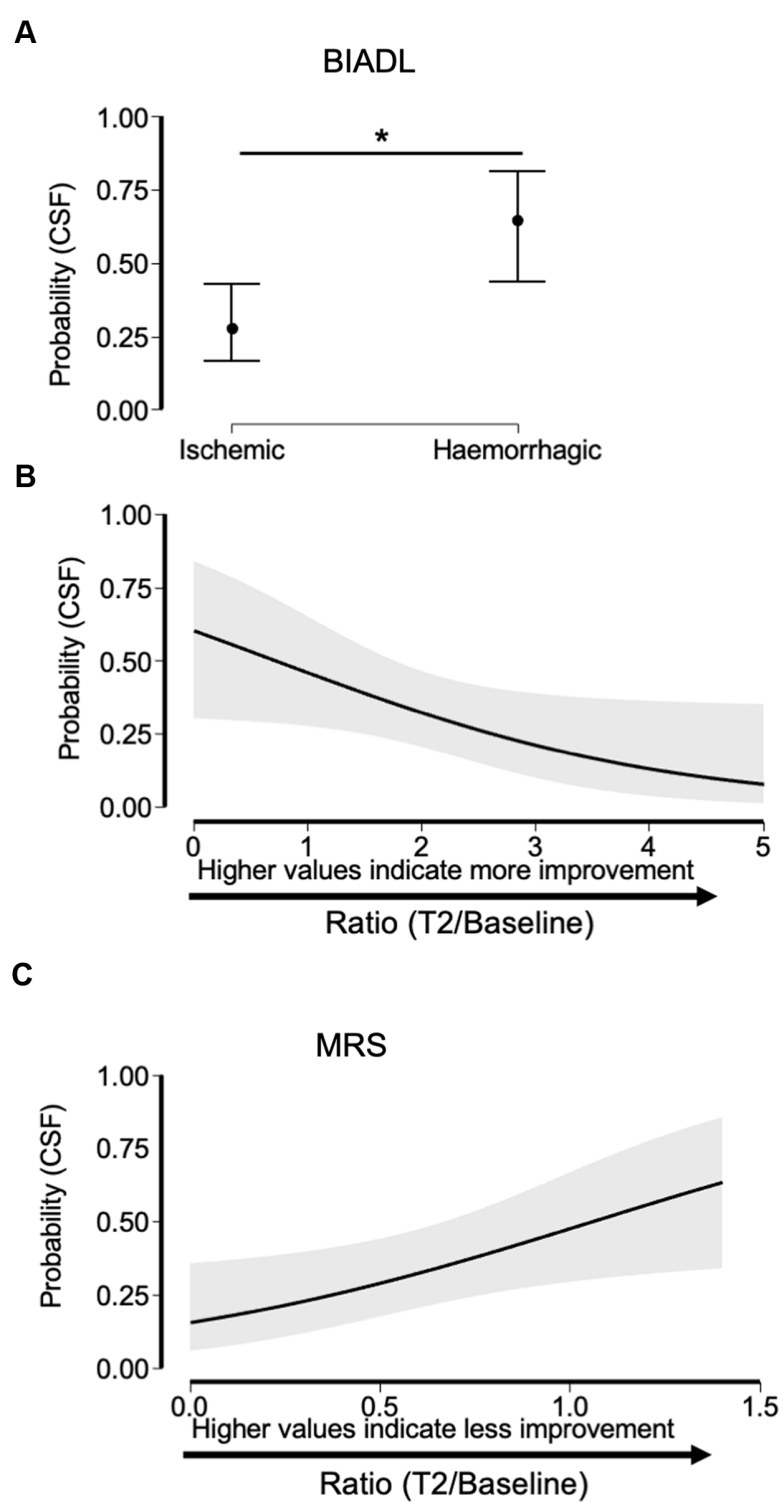
Relationship between having CSF at baseline with the stroke group (hemorrhagic or ischemic) and the improvement in the variables BIADL and MRS 6 months after stroke (T2). As BIADL concerns, the probability of having CSF is higher in hemorrhagic stroke **(A)**, so the factor hemorrhagic group is a significant risk factor for suffering CSF. The binary logistic regression model suggests a significant relationship between the CSF and BIADL. Higher BIADL values are associated with lower probabilities of having CSF at baseline **(B)**. The binary logistic regression model suggests a significant relationship between the CSI and MRS in the stroke group. Higher MRS values are associated with higher probabilities of having CSF **(C)**.

The results of the binary logistic regression analysis (including normalized NIHSS variable at baseline as a severity covariate) demonstrated a significant association between the presence of CSF at baseline and improvement of BIADL at T2 (OR = 0.560, Z = −1,982, CI = 0.315–0.994, *p* = 0.047; Figure 3), thus finding hemorrhagic stroke group as a significant risk factor for the presence of CSF (OR = 4.734, Z = 2.572, CI = 1.447–15.482, *p* = 0.010). In terms of the MRS, accounting for the normalized NIHSS variable at baseline as a severity covariate, a significant association was observed between the presence of CSF at baseline and improvement of MRS at T2 was observed (OR = 5.008, Z = 2.245, CI = 1.407–22.844, *p* = 0.015; see Figure 3). In this relationship, the stroke group does not contribute any significant risk factor as it was not even included in the resulting model. No significant association between the improvement of NIHSS and initial CSF was observed (*p* > 0.05). Moreover, none of the significant models included the NIHSS at baseline as a variable describing them.

## 4 Discussion

This study represents the first attempt to prospectively assess whether poststroke fatigue correlates with functional outcomes following a subacute rehabilitation period. Our findings revealed that poststroke clinically significant fatigue (CSF) was present in approximately 40% of patients at baseline, with a higher proportion observed in hemorrhagic stroke compared to ischemic stroke cases. Notably, the binary logistic regression analysis confirmed the predictive value of CSF at baseline on functional recovery and outcomes 6 months poststroke (19). Patients presenting with CSF at baseline demonstrated a higher probability of experiencing poor recovery.

Regarding outcome measurements obtained using the Barthel Index for Activities of Daily Living (BIADL), it appears that this probability varies between ischemic and hemorrhagic strokes, with the early presence of CSF being a risk factor for impaired improvement in hemorrhagic stroke. Despite similar improvements in functional measures (NIHSS, BIADL, and MRS) among ischemic and hemorrhagic patients, fatigue exhibited less improvement during the rehabilitation period in ischemic stroke cases, while significant improvement was observed in the hemorrhagic stroke group. This discrepancy cannot solely be attributed to initial stroke severity (which was the worst in the hemorrhagic group) or age and sex distribution (similar in both groups), suggesting potential differences in how the two stroke types affect the brain and potentially the networks governing fatigue (2–4, 11). In ischemic and hemorrhagic strokes, the processes of tissue damage, damage size and location, edema, inflammation, and blood not yet reabsorbed may be different and/or have a different time course, and this may cause different perceptions of fatigue. The specific mechanisms underlying fatigue induction in different stroke populations warrant further investigation (15).

Comorbidities such as hypertension, diabetes mellitus, depression, pain, and medication usage may independently contribute to fatigue (20–23). However, due to the limited sample size of our study, a formal analysis of these factors’ impact on our results was not conducted. While there is evidence suggesting an interrelationship between depressive mood, pain, and fatigue, indicating a symptom cluster, this does not necessarily imply shared aetiologies. Instead, it indicates that these factors are not entirely independent and warrant further exploration with larger cohorts to discern their individual effects on functional outcomes (10, 13, 20–26).

## 5 Study limitations

This study is subject to several limitations: (1) medications commonly prescribed for stroke patients, including sedatives, antiepileptics, pain medications, and antidepressants, may independently contribute to fatigue and may be differentially prescribed between ischemic and hemorrhagic strokes; (2) exclusion of patients unable to communicate their symptomatology, such as those with aphasia or altered states of consciousness (10, 26–28); (3) other conditioning factors, such as neuroanatomical/biological aspects and unexamined comorbidities, likely influence the effects of fatigue on functional outcomes, particularly the lack of improvement in fatigue despite neurorehabilitation in ischemic strokes (15–17, 19, 28). Our data confirm the evolution of poststroke fatigue and associated factors reported elsewhere (25, 29–31).

In conclusion, our data support the notion that early poststroke fatigue negatively impacts future outcomes and exhibits a distinct time course between ischemic and hemorrhagic strokes. Further studies are warranted to validate our findings and enhance their generalizability. Therefore, it is imperative to devise strategies during the acute and subacute phases poststroke to mitigate this detrimental effect and improve patient functions and quality of life.

## Data availability statement

The raw data supporting the conclusions of this article will be made available by the authors, without undue reservation.

## Ethics statement

The studies involving humans were approved by Hospital Universitario Severo Ochoa, Madrid, ref.: A1434. The studies were conducted in accordance with the local legislation and institutional requirements. The participants provided their written informed consent to participate in this study.

## Author contributions

AJ-B: Conceptualization, Data curation, Investigation, Methodology, Writing – original draft, Writing – review & editing. VS-L: Conceptualization, Data curation, Formal analysis, Investigation, Writing – review & editing. MD: Conceptualization, Investigation, Methodology, Validation, Writing – review & editing. EO: Investigation, Writing – review & editing. NL-A: Conceptualization, Investigation, Methodology, Writing – review & editing. AM: Writing – review & editing. JT: Formal analysis, Funding acquisition, Methodology, Writing – review & editing. AO: Conceptualization, Data curation, Methodology, Supervision, Validation, Writing – original draft, Writing – review & editing.
